# SARI suppresses colitis‐associated cancer development by maintaining MCP‐1‐mediated tumour‐associated macrophage recruitment

**DOI:** 10.1111/jcmm.14699

**Published:** 2019-10-02

**Authors:** Lei Dai, Yi Liu, Yuan Yin, Junshu Li, Zhexu Dong, Na Chen, Lin Cheng, Huiling Wang, Chao Fang, Yi Lin, Gang Shi, Hantao Zhang, Ping Fan, Xiaolan Su, Shuang Zhang, Yang Yang, Lie Yang, Wei Huang, Zongguang Zhou, Dechao Yu, Hongxin Deng

**Affiliations:** ^1^ State Key Laboratory of Biotherapy and Cancer Center West China Hospital Sichuan University and Collaborative Innovation Center for Biotherapy Chengdu China; ^2^ Department of Gastrointestinal Surgery West China Hospital and State Key Laboratory of Biotherapy Sichuan University Chengdu China; ^3^ Department of Clinical Research Management West China‐Liverpool Biomedical Research Center West China Hospital West China Biobanks Sichuan University Chengdu China; ^4^ Cancer Center West China Hospital Sichuan University Chengdu China

**Keywords:** colitis‐associated cancer, MCP‐1, SARI, STAT1, tumour‐associated macrophages

## Abstract

SARI (suppressor of AP‐1, regulated by IFN) impaired tumour growth by promoting apoptosis and inhibiting cell proliferation and tumour angiogenesis in various cancers. However, the role of SARI in regulating tumour‐associated inflammation microenvironment is still elusive. In our study, the colitis‐dependent and ‐independent primary model were established in SARI deficiency mice and immuno‐reconstructive mice to investigate the functional role of SARI in regulating tumour‐associated inflammation microenvironment and primary colon cancer formation. The results have shown that SARI deficiency promotes colitis‐associated cancer (CAC) development only in the presence of colon inflammation. SARI inhibited tumour‐associated macrophages (TAM) infiltration in colon tissues, and SARI deficiency in bone marrow cells has no observed role in the promotion of intestinal tumorigenesis. Mechanism investigations indicated that SARI down‐regulates p‐STAT1 and STAT1 expression in colon cancer cells, following inhibition of MCP‐1/CCR2 axis activation during CAC development. Inverse correlations between SARI expression and macrophage infiltration, MCP‐1 expression and p‐STAT1 expression were also demonstrated in colon malignant tissues. Collectively, our results prove the inhibition role of SARI in colon cancer formation through regulating TAM infiltration.

## 1 INTRODUCTION

According to the dynamic nature of the tumour microenvironment during malignant developing and progression, increasing interest has been focused on the role and mechanism of tumour microenvironment during tumour formation.^1^, ^2^ It is widely acknowledged that tumour microenvironment is an important regulator during tumour initiation, progression and metastasis.^3^, ^4^ Thus, imaging and shaping tumour microenvironment are a potential strategy for improving immunotherapy effect, drug delivery and chemo‐sensitivity.^5^, ^6^, ^7^

Inflammatory cells are a key component of the tumour microenvironment, and macrophages are present in almost all tumours. Through the production of inflammatory molecules such as interleukin‐6 (IL‐6), tumour necrosis factor‐α (TNF‐α) and interferon‐γ (IFN‐γ), macrophages play a crucial role in the tumour microenvironment.^8^ Substantial evidence indicates that macrophages adopt a pro‐oncogenic phenotype by promoting immunosuppressive tumour microenvironment formation, tumour cell invasion and angiogenesis.^9^, ^10^ Macrophages additionally produce growth factors and/or cytokines and recruit more inflammatory cells which would promote tumour initiation and progression.^8^, ^11^ Thus, targeting macrophage, including inhibition of macrophage recruitment to and/or survival in tumours, functional ‘re‐education’ of tumour associated macrophages (TAMs) to an anti‐tumour is an efficient strategy for cancer treatment.^10^

SARI (suppressor of AP‐1, regulated by IFN) was first characterized as a tumour suppressor in malignant glioma, melanoma and prostate cancer cells.^12^ In breast cancers and melanoma, SARI inhibits the transcription of CCN1 through blocking AP‐1 activity.^13^ Furthermore, SARI expression is necessary for mda‐7/IL‐24 anti‐tumour effects in breast cancer, melanoma, glioma cancers and prostate cancers.^14^ The previous study by us demonstrated that SARI inhibiting tumour angiogenesis and colon tumour growth through directly targeting ceruloplasmin and inhibiting the HIF‐1α/VEGF axis.^15^ A recent study by us also determined the anti‐tumour role of SARI‐targeted demethylation system by inhibiting tumour proliferation, angiogenesis and promoting apoptosis in colon cancer.^16^ However, the role of SARI in tumour‐associated inflammation microenvironment is still elusive.

In the present study, the colitis‐dependent and ‐independent primary model was established in SARI deficiency mice and immuno‐reconstructive mice to investigate the functional role of SARI in regulating tumour‐associated inflammation microenvironment and primary colon cancer development. Our results aimed to investigate the potential role of SARI in TAM infiltration and colitis‐associated cancer formation.

## 2 MATERIALS AND METHODS

### 2.1 Mice

The SARI wild‐type (SARI^WT^, catalog no. 002 448), SARI‐deficient (SARI^−/−^, catalog no. 019085) and CCR2‐deficient (CCR2^−/−^, catalog no. 004999) mice were purchased from Jackson Laboratory and kept in Specific Pathogen Free room at 25°C under a 12‐hours light/dark cycle, with ad libitum access to food and water. The SARI^−/−^CCR2^−/−^ strain was generated, and the mouse gene type was determined. All mouse care and studies were performed following the institutional guidelines concerning animal use and care of Sichuan University.

### 2.2 Induction of colitis‐associated cancer

Mice were injected intraperitoneally (i.p.) with 10 mg/kg azoxymethane (AOM; Sigma) and after 7 days, received 2% DSS for 3 cycles (7 days for 2% DSS and 14 days with normal drinking water). At 63 days post‐AOM injection, high resolution mini‐endoscopy (STOKE, Germany) was performed to detect the degree of CAC formation before mice were sacrificed. Colons were removed and flushed with PBS, and tumours were counted and measured with calipers.

### 2.3 Induction of colitis‐independent colon cancer

Mice were injected intraperitoneally (i.p.) with 10 mg/kg azoxymethane (AOM; Sigma) six times with one week intervals. At 20 weeks post‐AOM injection, high resolution mini‐endoscopy (STOKE, Germany) was performed to detect the degree of CAC formation before mice were sacrificed. Colons were removed and flushed with PBS, and tumours were counted and measured with calipers.

### 2.4 Histopathological analysis

The colon tissues were collected and embedded by paraffin. After cutting into 4 μm slides, H&E staining kit (C0105, Beyotime) was employed to detect the histomorphology of the colon tissues. Image J software was employed to calculate the per cent of tumour area based on the H&E staining after designating the tumour area by a pathologist. Immunohistochemical (IHC) staining was performed with IHC staining kit (SP9001, SP9002, Zsbio). Briefly speaking, after dewaxing, antigen retrieval was performed under high temperature and high pressure for 3 minutes. Then, the hydrogen peroxide and goat serum were used to treat the slides at room temperature for 15 times, interval with PBS washing for 3 times. The primary antibodies against PCNA, CD68, MCP‐1, p‐STAT1, F4/80, iNOS and CCR2 were added and incubated for overnight at 4˚C. After incubation with the suitable secondary antibodies, DAB kit (maixin, Fuzhou, China) was performed to detect the positive cells. Then, the cell nucleus was stained by haematoxylin (Beyotime, Beijing, China). The positive cells and total cells in the same frame were counted and used to calculate the percentage of positive cells. For immunofluorescence staining, the sections were incubated with fluorescein isothiocyanate‐conjugated and tetramethylrhodamine‐conjugated secondary antibodies (Thermo Fisher, MA, USA) and the nucleus was stained with 4',6‐diamidino‐2‐phenylindole (DAPI, Thermo Fisher). Apoptosis was detected in paraffin‐embedded colon samples using the DNA Fragmentation Detection Kit (QIA39, Merck) according to the manufacturer's instructions. Positive cells in each frame were counted for further analysis. An Olympus BX51 upright microscope was used to take the images using appropriate fluorescence filters and white filters.

### 2.5 Bone marrow chimeras

The mice of bone marrow chimeras were established as the previous study indicated.^17^ Briefly speaking, SARI^WT^ and SARI^−/−^ mice (8‐week‐old) were γ‐irradiated with 7.0 Gray (3.5 Gray each time, with a three‐hour interval) using the RS‐200 Biological Irradiator. 2 × 10^6^ BM cells, which isolated from SARI^WT^ and SARI^−/−^ mice, were intravenously injected into the irradiated mice. The gene type determinate at 8 weeks after BM injection, based on the blood. The defined mice were used for further experiential study.

### 2.6 Flow cytometry

Flow cytometry in the present study was followed with the procedure of the previous study by us.^18^ The inflammatory cells in colonic tissues were detected by primary antibodies against CD45 (cat. no. 103 116), CD11b (cat. no. 101 228), F4/80 (cat. no. 123 108), NK1.1 (cat. no. 108 710), CD19 (cat. no. 115 507), Ly6G (cat. no. 127 623) and CD11c (cat. no. 117 308) which were obtained from BioLegend and CD4 (cat. no. 553 030) and CD8 (cat. no. 100 722), which were obtained from BD Bioscience. The flow cytometry was analysed by NovoCyte flow cytometer (ACEA Biosciences) and the data were analysed with NovoExpress software (ACEA Biosciences).

### 2.7 Western blotting

After the determination of protein concentration by the BCA kit (Thermo Fisher), the total of 20 μg of protein was loaded into lanes and separated by SDS–PAGE gel electrophoresis. After transferring proteins onto polyvinylidene difluoride (PVDF) membranes (Merck Millipore), the membrane was blocked with TBS/T buffer containing 5% milk at room temperature for 1 hour. The primary antibodies against SARI (1:400, Abcam), p‐STAT1 (1:1000, CST), STAT1 (1:1200, CST), p‐c‐Jun (1:1000, CST), p‐STAT3 (1:800, CST), STAT3 (1:1200, CST), NF‐κB (1:1000, CST), IKKβ (1:1200, CST), NF‐κB p105 (1:1200, CST), p‐IκBα (1:1200, CST), HA (1:2000, CST) and GAPDH (1:50,000, CST) were added to detect the specific band. Chemiluminescent substrate ECL kit (Merck Millipore) was used to detect the bands.

### 2.8 Enzyme‐linked immunosorbent assay

The total proteins of the entire colon of mice were extracted by RIPA lysis buffer (Beyotime, Nanjing, China) containing 1% protease inhibitor cocktail (Merck Millipore). After determination of the protein concentration by BCA kit (Thermo Fisher), the expression of mouse MCP‐1, IL‐6, TNF‐α, IL‐1β and IFN‐γ was determined by the ELISA kits from NeoBioscience (Shenzhen, China).

### 2.9 Real‐time PCR

Total RNA was extracted from colon tissues following the instructions of TRIzol reagent (Invitrogen). After the determination of RNA concentration by ND2000 (Thermo Fisher), 1 μg of RNA samples was used to generate cDNA using the TaKaRa RT reagent kit from Takara. Real‐time PCR was performed on Light Cycler 96 (Roche) with SYBR Green kit (Takara, Tokyo, Japan). Relative mRNA expression was calculated by 2^−ΔΔCt^, after normalization to the levels of U6 mRNA.

### 2.10 Cell culture and treatment

SW480 and HCT116 cells were purchased from the American Type Culture Collection (VA, USA) and cultured in DMEM (Invitrogen, MA, USA) containing 10% foetal bovine serum and antibiotics. The lentivirus‐based SARI overexpression system and control system were used to infect SW480 and HCT116 cells separately. After adding puromycin for selecting, the stably infected cells were named as SW480‐Ctrl, SW480‐SARI, HCT116‐Ctrl and HCT116‐SARI.

### 2.11 Clinical samples and analysis

Twenty colon cancer tissues (eight from male patients and 12 from female patients) were collected at the West China Hospital (Chengdu, China) following the inclusion criteria: age 60‐70, TNM stage II‐III, histological grade II‐III. All of the protocol was approved by the Ethics Committee of West China Hospital. After embedded with paraffin, the 5 μm sections were used to determine SARI, CD68, MCP‐1 and p‐STAT1 expression by immunohistochemistry. Tumours were scored based on the positive cells in each slide. The correlation between SARI, CD68, MCP‐1 and p‐STAT1 expression in human colon cancer tissue was analysed based on the immunohistochemistry score.

### 2.12 Statistical analysis

All of the data were presented as the mean ± standard deviation, and the statistical analysis was performed using SPSS v17.0 (SPSS Inc, Chicago, IL, USA). The Student's *t* test was used for comparing two groups, and analysis of variance was used for multiple group comparisons. The Pearson correlation analysis was performed to determine the correlation between the groups. *P* < .05 was considered statistically significant.

## 3 RESULTS

### 3.1 SARI deficiency promotes colon tumorigenesis only in the presence of colon inflammation

**Figure 1:**
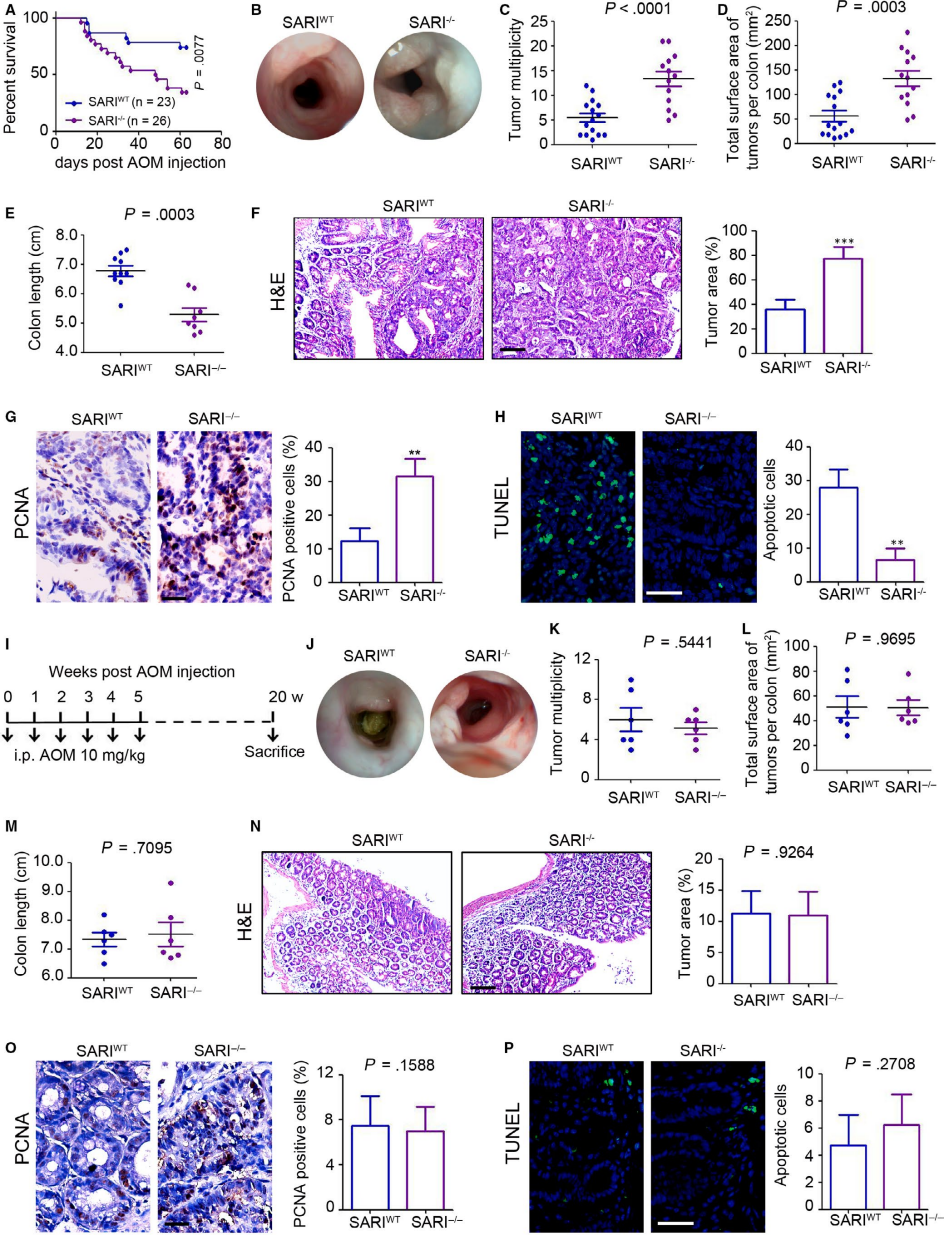
SARI deficiency promotes colon tumorigenesis only in the presence of colon inflammation. (A) Survival analysis of SARI^WT^ and SARI^−/−^ mice after receiving AOM (i.p. injection, 10 mg/kg) and DSS (2% in drinking water, three cycles) treatment for 63 d. (n = 23 for SARI^WT^ mice and n = 26 for SARI^−/−^ mice; *P* = .0077, long‐rank test). (B) High resolution mini‐endoscopy images of colon cross‐sections at the end of AOM/DSS treatment. (C‐E) Tumour multiplicity (C), total tumour surface area (D) and colon length (E) of per mice at the end of AOM/DSS treatment. Data represent means ± SD, Student's *t* test. (F) Representative images of H&E staining of colon tumours at the end of the AOM/DSS treatment. The tumour area was microscopically analysed. Scale bar = 100 μm, ***P < .001, Student's *t* test. (G) Representative images of PCNA staining of colon tumours at the end of the AOM/DSS treatment. The per cent of PCNA positive cells were microscopically analysed. Scale bar = 50 μm, **P < .05, Student's *t* test. (H) Representative images of TUNEL staining of colon tumours at the end of the AOM/DSS treatment. The per cent of apoptotic cells (TUNEL positive) were microscopically analysed. Scale bar = 100 μm, **P < .05, Student's *t* test. (I) Schematic overview of the AOM‐induced (i.p. injection, 6 times) colon tumours. Mice were sacrificed for analysis at 20 wk post‐AOM injection. (J) High resolution mini‐endoscopy images of colon cross‐sections at the end of 6 × AOM treatment. (K‐M) Tumour multiplicity (K), total tumour surface area (L) and colon length (M) of per mice at the end of 6 × AOM treatment. Data represent means ± SD, Student's *t* test. (N) Representative images of H&E staining of colon tumours at the end of the 6 × AOM treatment. The tumour area was microscopically analysed. Scale bar = 100 μm. (O) Representative images of PCNA staining of colon tumours at the end of the 6 × AOM treatment. The per cent of PCNA positive cells were microscopically analysed by Student's *t* test. Scale bar = 50 μm. (Q) Representative images of TUNEL staining of colon tumours at the end of the 6 × AOM treatment. The per cent of apoptotic cells (TUNEL positive) were microscopically analysed. Data represent means ± SD, Student's *t* test. Scale bar = 100 μm

To investigate the potential role of SARI in primary colon cancer initiation and progression, azoxymethane (AOM)/dextran sodium sulphate (DSS) model of CAC was established (Figure S1A) in mice. We found colonic SARI expression was down‐regulated after 2% DSS treatment both at the mRNA (Figure S1B) and the protein levels (Figure S1C). Furthermore, SARI wild‐type (*SARI* ^WT^) and *SARI* knockout (*SARI* ^−/−^) mice were applied for the CAC model as shown in Figure S1A. Higher mortality was observed in SARI^−/−^ mice with CAC (Figure 1A). In vivo high resolution mini‐endoscopy imaging indicated an increased tumour load in *SARI* ^−/−^ mice compared with that in *SARI* ^WT^ mice, accompanied with markedly increased tumour multiplicity (Figure 1C) and tumour size (Figure 1D). There was a significant decrease in colon length in *SARI* ^−/−^ mice (Figure 1E). H&E staining of colonic tissues likewise indicated a greater tumour area in *SARI* ^−/−^ mice following AOM/DSS treatment (Figure 1F). Notably, more proliferative and less apoptotic cells were detected by proliferating cell nuclear antigen (PCNA) staining (Figure 1G) and terminal deoxynucleotidyl transferase dUTP nick end labelling (TUNEL) assay (Figure 1H) in *SARI* ^−/−^ mice.

Next, the primary colon cancer model in the absence of tissue inflammation was established by repeated injections with AOM (six times, 10 mg/kg, one injection per week, Figure 1I). There was no dramatic difference in tumour load, tumour multiplicity, tumour area and colon length between *SARI* ^WT^ and *SARI* ^−/−^ mice (Figure 1J‐M). H&E staining additionally showed similar results in intestinal tumorigenesis (Figure 1N). No changes in the numbers of proliferative and apoptotic cells in colonic tissues were observed in *SARI* ^−/−^ mice in the 6 × AOM injection model (Figure 1 O and P). Collectively, these data suggested that colon inflammation is necessary for the deletion of SARI to promote intestinal tumorigenesis.

### 3.2 SARI impairs colon damage during CAC development in mice

**Figure 2:**
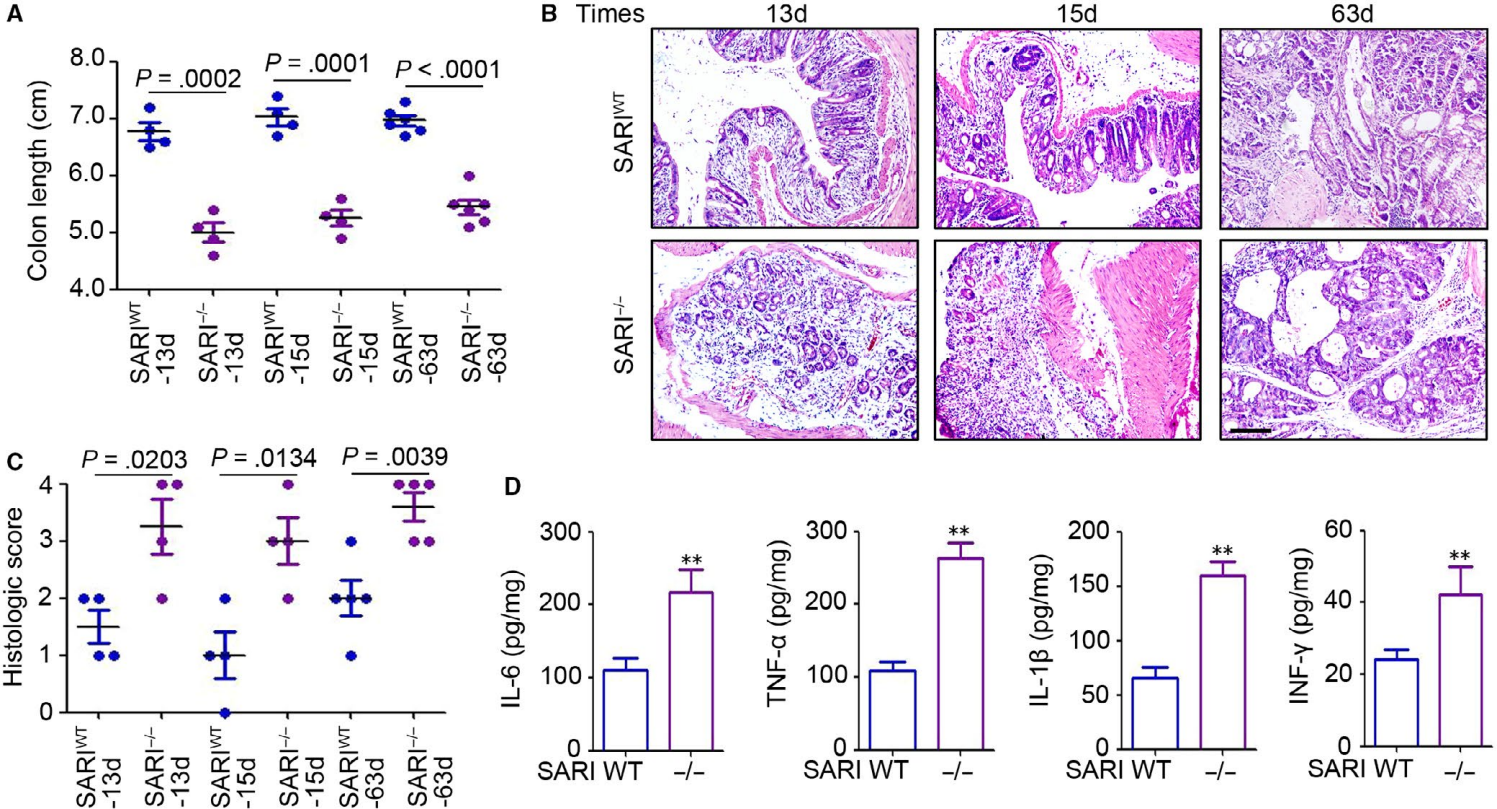
SARI deficiency promotes colitis during CAC. (A) Colon length of SARI^WT^ and SARI^−/−^ mice after receiving AOM injection for 13 d, 15 d and 63 d in AOM/DSS model. Data represent means ± SD, analysis of variance. (B&C) Representative images of H&E staining of colon tissue at 13 d, 15 d and 63 d after AOM injection in the AOM/DSS model. The histological inflammation was microscopically analysed. Data represent means ± SD, analysis of variance. (D) Cytokines expression in whole colons from SARI^WT^ and SARI^−/−^ mice after receiving AOM injection for 63 d in the AOM/DSS model were determined by ELISA. (n = 4, **, *P* < .01, Student's *t* test)

To define the role of SARI in colitis‐associated inflammation, *SARI* ^WT^ and *SARI* ^−/−^ mice with AOM/DSS treatment were sacrificed at 13, 15 and 63 days post‐AOM injection. We demonstrated that the colon length, an obvious indicator of inflammation, in *SARI* ^−/−^ mice, was significant decreased (Figure 2A) at 13, 15 and 63 days post‐AOM injection, accompanied with higher severity of inflammation, evidenced by inflammatory cells infiltration and loss of goblet cells (Figure 2B and C). Notably, higher expression of pro‐inflammatory cytokines (IL‐6, TNF‐α, IL‐1β and IFN‐γ; Figure 2D) was found in *SARI* ^−/−^ mice compared with that in SARI^WT^ mice at the 63 days post‐AOM treatment. The above data indicated that *SARI* deficiency mice are hyper susceptible to colitis during CAC development.

### 3.3 SARI deficiency promotes TAM recruitment in colon tissue

**Figure 3:**
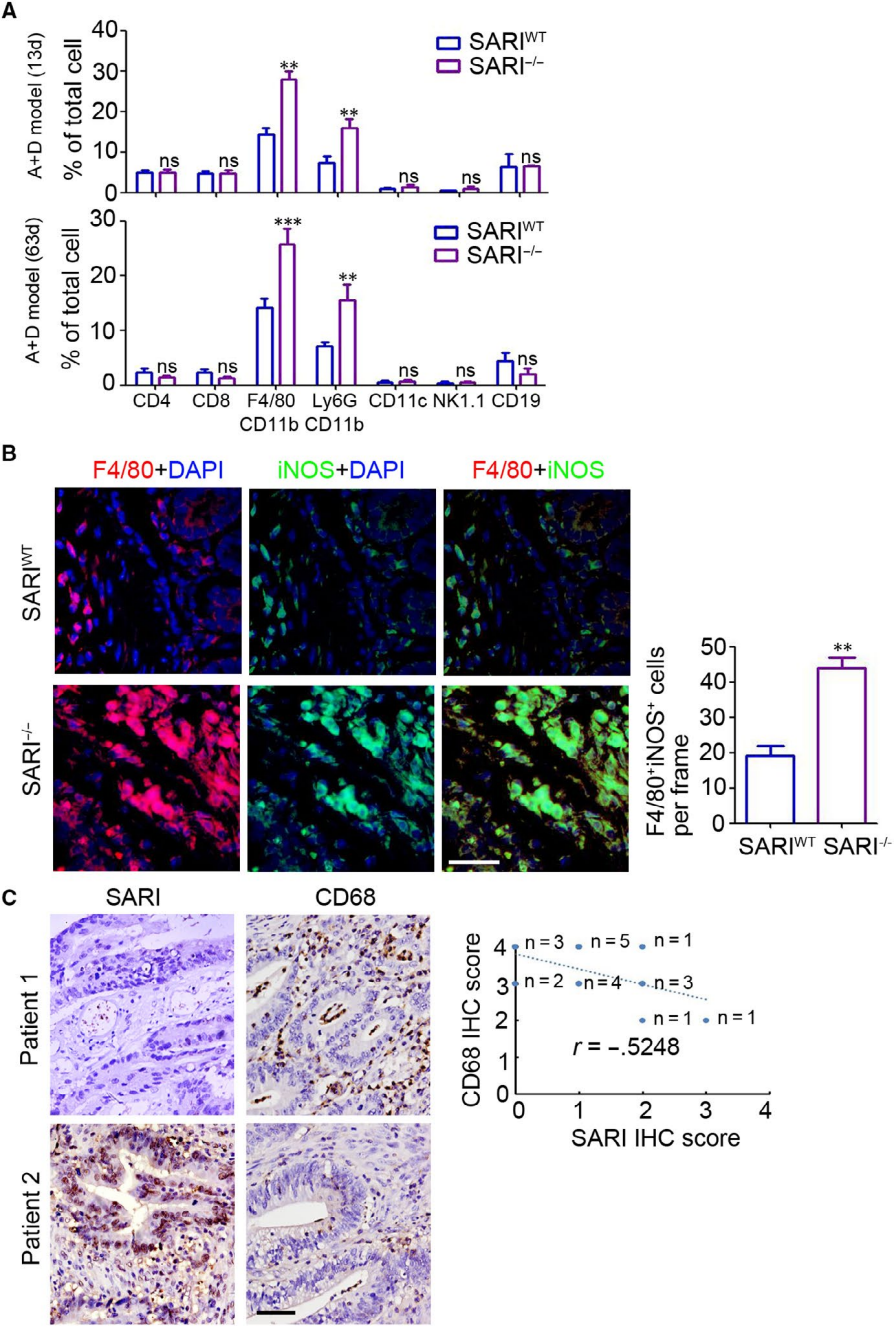
SARI inhibits TAM recruitment in CAC. (A) Flow cytometry analysis of immune cells (CD4^+^ T cells, CD8^+^ T cells, CD11b^+^F4/80^+^ macrophages, CD11b^+^Ly6G^+^ MDSC, CD11c^+^ DC, NK1.1^+^ NK cells and CD19^+^ B cells) infiltration in colonic tissues of SARI^WT^ and SARI^−/−^ mice at 13 d and 63 d post‐AOM injection in AOM/DSS model. Data represent means ± SD, Student's *t* test. (B) Representative images of F4/80 (red) and iNOS (green) staining of colon tissues at the end of the AOM/DSS treatment. The F4/80 and iNOS positive cells were microscopically analysed. Scale bar = 50 μm, **, *P* < .001, Student's *t* test. (C) Representative images of SARI and CD68 staining of colon tumours from patients. The correlation between SARI and CD68 in colon tumours was analysed. Scale bar = 100 μm. (n = 20, *r* = −0.5248, Pearson correlation analysis)

To determine the underlying effect of SARI on the inflammatory microenvironment during regulating intestinal tumorigenesis, flow cytometry was employed to determine the leucocyte infiltration. Significant increasing of macrophages (F4/80^+^/CD11b^+^) and neutrophils (Ly6G^+^/CD11b^+^) was found in the colon sections of SARI^−/−^ mice at 13 days and 63 days post‐AOM treatment, compared with those in SARI^WT^ mice (Figure 3A). Immunofluorescence staining also confirmed the increasing infiltration of iNOS positive macrophages in the colon sections of SARI^−/−^ mice compared with that in SARI^WT^ mice when the CAC formed (63 days post‐AOM treatment, Figure 3B). To determine whether macrophages present in human colon tumour tissues are correlated with SARI expression, we used immunohistochemical (IHC) staining with anti‐SARI and anti‐CD68 antibodies to detect SARI protein expression and macrophages in 20 human colon tumour tissues and found there was an increase in macrophage infiltration in malignant tissues (Figure S2) and an inverse correlation between SARI expression and macrophage infiltration (Figure 3C). These results demonstrated the inhibitional role of SARI in TAM infiltration into colon sections during CAC development.

### 3.4 The effect of immune cells during SARI regulating CAC development

**Figure 4:**
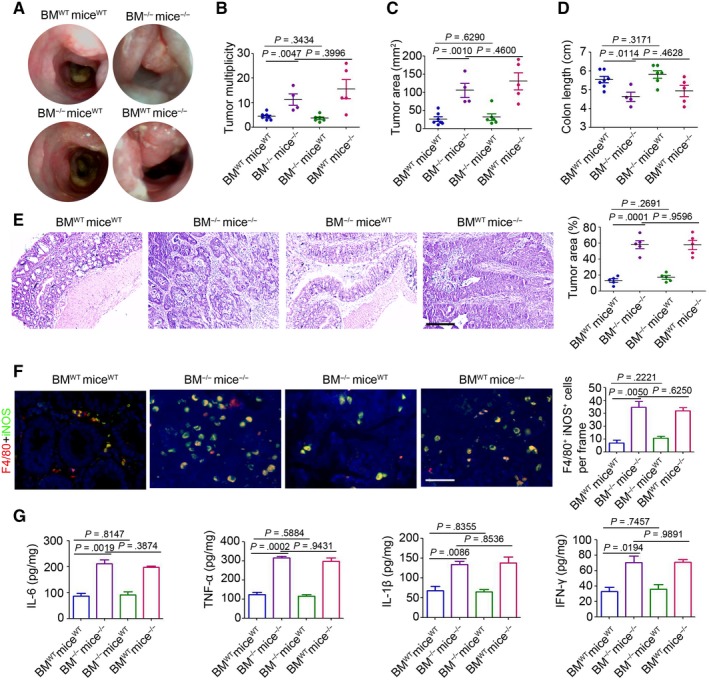
The effect of immune cells during SARI regulating CAC. (A) High resolution mini‐endoscopy images of the colons of SARI^WT^ mice with SARI^WT^ BM (BM^WT^mice^WT^), SARI^−/−^ mice with SARI^−/−^ BM (BM^−/−^ mice^−/−^), SARI^WT^ mice with SARI^−/−^ BM (BM^−/−^mice^WT^) and SARI^−/−^ mice with SARI^WT^ BM (BM^−/−^mice^WT^) receiving AOM/DSS treatment (AOM, i.p. injection 10 mg/kg; DSS, 2% in drinking water for three cycles). (B‐D) Tumour multiplicity (B), tumour area (C) and colon length (D), of SARI^WT^ mice with SARI^WT^ BM (BM^WT^mice^WT^), SARI^−/−^ mice with SARI^−/−^ BM (BM^−/−^ mice^−/−^), SARI^WT^ mice with SARI^−/−^ BM (BM^−/−^mice^WT^) and SARI^−/−^ mice with SARI^WT^ BM (BM^−/−^mice^WT^) receiving AOM/DSS treatment (AOM, i.p. injection 10 mg/kg; DSS, 2% in drinking water for three cycles). Data represent means ± SD, analysis of variance. (E) H&E images of colon cross‐section and the quantitation of histological tumour area of SARI^WT^ mice with SARI^WT^ BM (BM^WT^mice^WT^), SARI^−/−^ mice with SARI^−/−^ BM (BM^−/−^ mice^−/−^), SARI^WT^ mice with SARI^−/−^ BM (BM^−/−^mice^WT^) and SARI^−/−^ mice with SARI^WT^ BM (BM^−/−^mice^WT^) AOM/DSS treatment (AOM, i.p. injection 10 mg/kg; DSS, 2% in drinking water for three cycles). Data represent means ± SD, analysis of variance. Scale bar = 200 μm. (F) Representative images of F4/80 (red) and iNOS (green) staining of colon tissues of SARI^WT^ mice with SARI^WT^ BM (BM^WT^mice^WT^), SARI^−/−^ mice with SARI^−/−^ BM (BM^−/−^ mice^−/−^), SARI^WT^ mice with SARI^−/−^ BM (BM^−/−^mice^WT^) and SARI^−/−^ mice with SARI^WT^ BM (BM^−/−^mice^WT^) receiving AOM/DSS treatment (AOM, i.p. injection 10 mg/kg; DSS, 2% in drinking water for three cycles). The cell nucleus was stained with DAPI (blue). The F4/80 and iNOS positive cells were microscopically analysed. Data represent means ± SD, analysis of variance. Scale bar = 100 μm, **, *P* < .01. (G) Cytokines expression in whole colons from SARI^WT^ mice with SARI^WT^ BM (BM^WT^mice^WT^), SARI^−/−^ mice with SARI^−/−^ BM (BM^−/−^ mice^−/−^), SARI^WT^ mice with SARI^−/−^ BM (BM^−/−^mice^WT^) and SARI^−/−^ mice with SARI^WT^ BM (BM^−/−^mice^WT^) receiving AOM/DSS treatment (AOM, i.p. injection 10 mg/kg; DSS, 2% in drinking water for three cycles). (n = 4, **P < .01; ns, no significant difference, compared with BM^WT^mice^WT^ group). Data represent means ± SD, analysis of variance

To investigate the effects of bone marrow (BM)‐derived macrophages in SARI^WT^ and SARI^−/−^ mice, the immuno‐reconstructed mice were constructed and performed for the CAC model. The results have shown that three out of the seven irradiated SARI^−/−^ mice receiving SARI^WT^ BM and two out the seven irradiated SARI^−/−^ mice receiving SARI^−/−^ BM died during the intestinal tumorigenesis process, while none of the irradiated SARI^WT^ mice receiving SARI^WT^ and SARI^−/−^ BM died (Figure S3A). The irradiated SARI^−/−^ mice receiving SARI^WT^ and SARI^−/−^ BM both showed more primary tumour formation (Figure 4A), accompanied with marked increase in tumour multiplicity (Figure 4B) and tumour size (Figure 4B) and a decrease in colon length (Figure 4D). Histological staining of colonic tissues indicated greater tumour area and more proliferative cells were observed in these immuno‐reconstructed mice (Figure 4E and Figure S3B). Notably, a marked increased colonic inflammation was detected in the irradiated SARI^−/−^ mice receiving SARI^WT^ and SARI^−/−^ BM, as evidenced by an increase in the number of activated macrophages (Figure 4F) and higher pro‐inflammatory cytokine expression (IL‐6, TNF‐α, IL‐1β and IFN‐γ) compared with those in the irradiated SARI^WT^ mice receiving SARI^WT^ and SARI^−/−^ BM (Figure 4G). These results further demonstrated that SARI deficiency in BM cells has no observed role in the promotion of intestinal tumorigenesis.

### 3.5 SARI deficiency promoted MCP‐1 production in CAC mice

**Figure 5:**
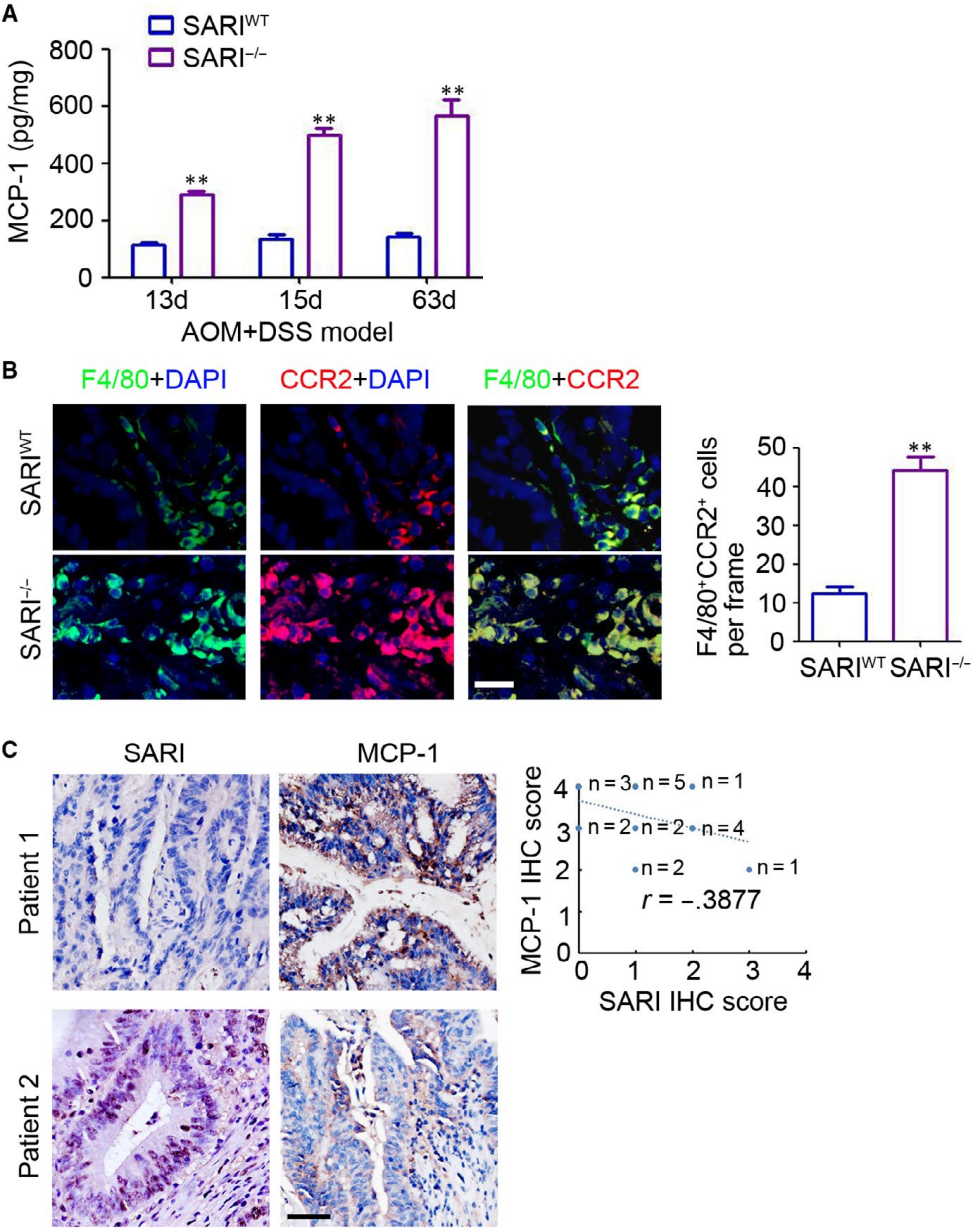
SARI inhibits MCP‐1 expression in CAC. (A) ELISA analysis of MCP‐1 expression in SARI^WT^ mice and SARI^−/−^ mice receiving AOM/DSS treatment for 13 d, 15 d and 63 d. (n = 4, **, *P* < .01, analysis of variance). (B) Representative images of F4/80 (green) and CCR2 (red) staining of colon tissues of SARI^WT^ mice and SARI^−/−^ mice receiving AOM/DSS treatment for 63 d. The cell nucleus was stained with DAPI (blue). The F4/80 and CCR2 positive cells were microscopically analysed. Scale bar = 60 μm, **P < .01, Student's *t* test. (C) Representative images of SARI and MCP‐1 staining of colon tumours from patients. The correlation between SARI and MCP‐1 in colon tumours was analysed. Scale bar = 100 μm. (n = 20, *r* = −0.3877, Pearson correlation analysis)

ELISA assays confirmed that MCP‐1 expression was maintained at a high level and markedly increased in SARI^−/−^ mice at 13, 15 and 63 days after AOM/DSS treatment (Figure 5A). Moreover, immunofluorescence analysis suggested that more CCR2 positive macrophages (F4/80^+^) were found in the CAC tissues of SARI^−/−^ mice (Figure 5B). Furthermore, histological examination demonstrated that MCP‐1 expression is up‐regulated in malignant tissues (Figure S4) and is inversely correlated with SARI expression in colon tumour tissues from patients (Figure 5C). These results demonstrated that SARI inhibits MCP‐1 expression during regulating CAC.

### 3.6 CCR2 plays a necessary role during SARI regulating CAC development

**Figure 6:**
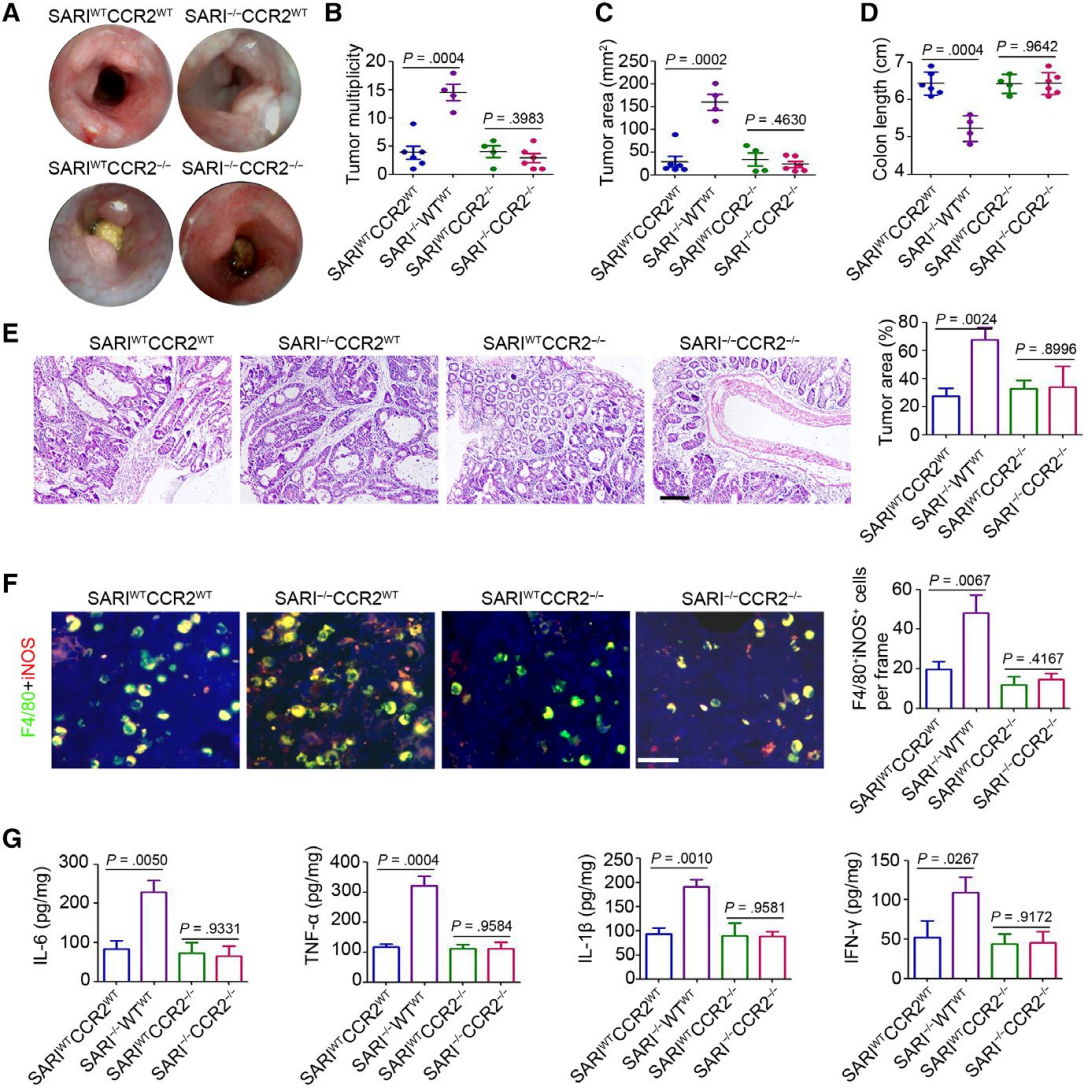
MCP‐1/CCR2 axis plays a necessary role during SARI regulating CAC. (A) High resolution mini‐endoscopy images of colon cross‐sections from SARI^WT^CCR2^WT^, SARI^−/−^CCR2^WT^, SARI^WT^CCR2^−/−^, SARI^−/−^CCR2^−/−^ mice at the end of AOM/DSS treatment. (B‐D) Tumour multiplicity (B), total tumour surface area (C) and colon length (D) of SARI^WT^CCR2^WT^, SARI^−/−^CCR2^WT^, SARI^WT^CCR2^−/−^, SARI^−/−^CCR2^−/−^ mice at the end of AOM/DSS treatment. Data represent means ± SD, analysis of variance. (E) Representative images of H&E staining of colon tumours from SARI^WT^CCR2^WT^, SARI^−/−^CCR2^WT^, SARI^WT^CCR2^−/−^, SARI^−/−^CCR2^−/−^ mice at the end of the AOM/DSS treatment. The tumour area was microscopically analysed. Scale bar = 100 μm, ***P < .001. Data represent means ± SD, analysis of variance. (F) Representative images of F4/80 (red) and iNOS (green) staining of colon tissues SARI^WT^CCR2^WT^, SARI^−/−^CCR2^WT^, SARI^WT^CCR2^−/−^, SARI^−/−^CCR2^−/−^ mice at the end of the AOM/DSS treatment. The cell nucleus was stained with DAPI (blue). The F4/80 and iNOS positive cells were microscopically analysed. Data represent means ± SD, analysis of variance. Scale bar = 100 μm. (G) Cytokines expression in whole colons from SARI^WT^CCR2^WT^, SARI^−/−^CCR2^WT^, SARI^WT^CCR2^−/−^, SARI^−/−^CCR2^−/−^ mice at the end of the AOM/DSS treatment (n = 4). Data represent means ± SD, analysis of variance

To further investigate the role of MCP‐1 during SARI‐regulated CAC, SARI^−/−^CCR2^−/−^ and single‐gene knockout mice were generated for the CAC model. The results indicated that CCR2 knockout blocks the SARI deficiency‐mediated tumorigenesis, as evidenced by representative mini‐endoscopic images (Figure 6A), tumour multiplicity analysis (Figure 6B), tumour area analysis (Figure 6C) and colon length analysis (Figure 6D). Histological analysis also confirmed the less tumour area in SARI^−/−^CCR2^−/−^ and SARI^WT^CCR2^−/−^ mice relative to SARI^−/−^CCR2^WT^ mice (Figure 6E). Moreover, fewer infiltrating activated macrophages and lower pro‐inflammatory expression were observed in SARI^−/−^CCR2^−/−^ and SARI^WT^CCR2^−/−^ mice at 63 days after AOM/DSS treatment (Figure 6F and G). Collectively, the above data indicated that CCR2 plays a necessary role during SARI regulating CAC development.

### 3.7 SARI directly targets and inhibits STAT1 expression in colon cancer cells

**Figure 7:**
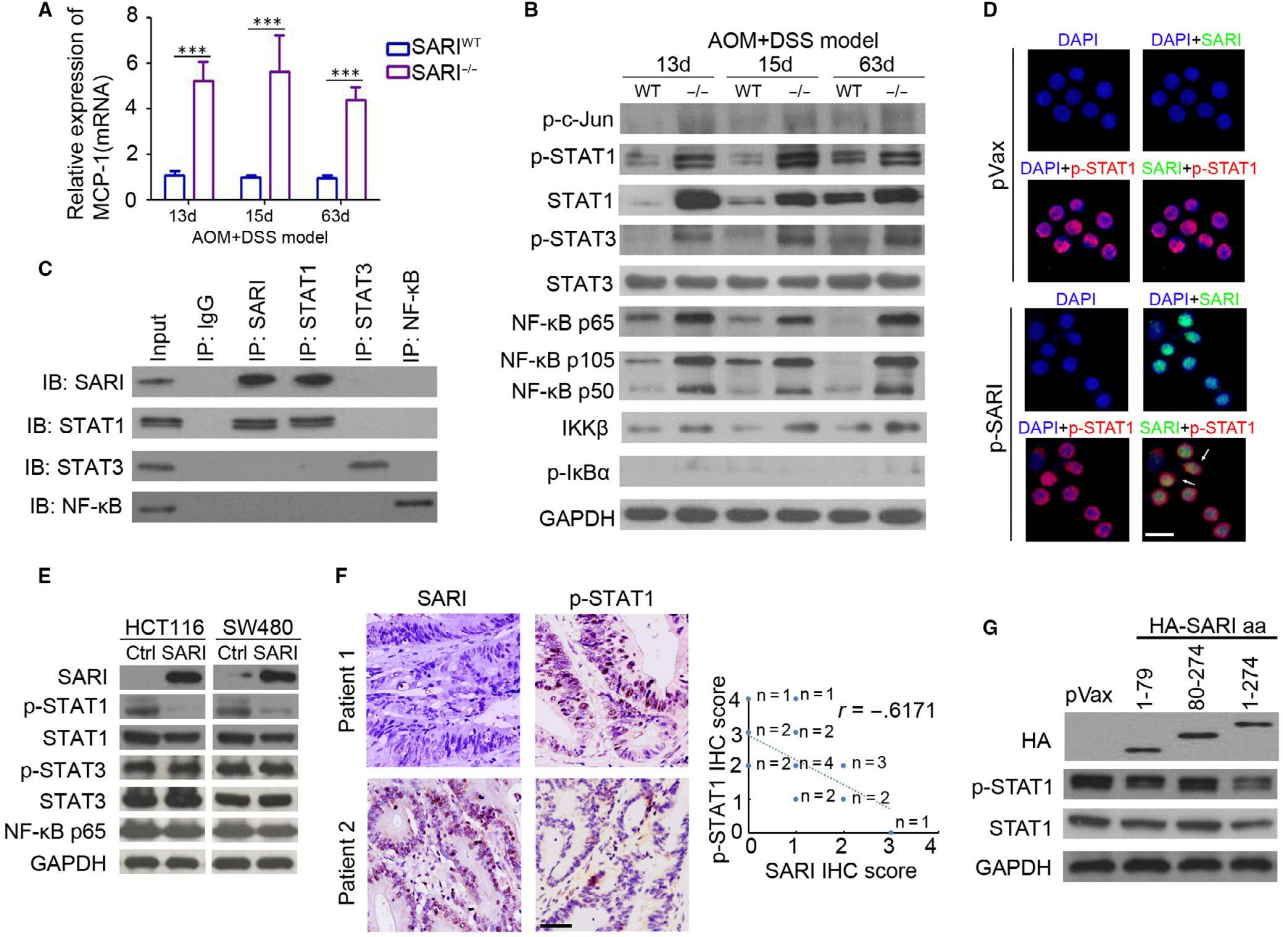
SARI directly targets and inhibits STAT1 expression in colon cancer cells. (A) Real‐time PCR analysis of MCP‐1 expression in whole colonic tissues from CAC mice. (n = 4, ***P < .0001, data represent means ± SD, analysis of variance). (B) Immunoblots of inflammation‐related protein expression in whole colonic tissues from CAC mice. (C) Co‐immunoprecipitation analysis in SW480‐SARI cells using anti‐SARI, anti‐STAT1, anti‐STAT3 and anti‐NF‐κB antibody. (D) Staining for SARI (green) and p‐STAT1 (red) in SW480 cells that transfected with p‐SARI or pVax empty plasmid; DAPI staining for the nucleus. Scale bar = 20 μm. (E) Immunoblots of SARI and inflammation‐related protein expression in HCT116‐control, SARI and SW480‐control, SARI cells. GAPDH was used as a loading control. (F) Representative images of SARI and p‐STAT1 staining of colon tumours from patients. The correlation between SARI and p‐STAT1 in colon tumours was analysed. Scale bar = 100 μm. (n = 20, *r* = −0.3877, Pearson correlation analysis). (G) Immunoblots of HA, p‐STAT1 and STAT1 expression in SW480 cells after transfection with pVax, HA‐SARI (encoding 1‐79 amino acids), HA‐SARI (encoding 80‐274 amino acids) and HA‐SARI (encoding 1‐274 amino acids). GAPDH was used as a loading control

As shown in Figure 7A, SARI inhibits MCP‐1 mRNA expression in CAC mice. Further results confirmed the up‐regulation of p‐STAT1, p‐STAT3 and NF‐κB expression and hyperactivation of the NF‐κB pathway in CAC from SARI^−/−^ mice (Figure 7B). Immunoprecipitation and western blotting showed that SARI specifically interacts with STAT1, but not STAT3 and NF‐κB in colon cancer cells (Figure 7C). Immunofluorescence analysis revealed that SARI (green) and p‐STAT1 (red) co‐localized in the nucleus of SW480 cells (Figure 7D). In an in vitro study, ectopic expression of SARI inhibited p‐STAT1 and STAT1 levels, but had no observed effect on STAT3 and NF‐κB expressions in colon cancer cells (Figure 7E). Further histological examination suggested that there is inverse correlation between SARI and p‐STAT1 level, which is frequently up‐regulated in colon tumour tissues from patients (Figure S5A), whereas SARI mRNA expression was positively correlated with STAT1 mRNA expression (Figure S5B). Moreover, SARI additionally down‐regulated INF‐β‐induced STAT1 activation in SW480 cells (Figure S5C). The stepwise deletion of SARI suggested that amino acids 1‐79 are crucial for reducing p‐STAT1 expression (Figure 7G). Collectively, the above results indicated that SARI directly targets STAT1 and inhibits p‐STAT1 and STAT1 expression in colon cancer cells.

**Figure 8:**
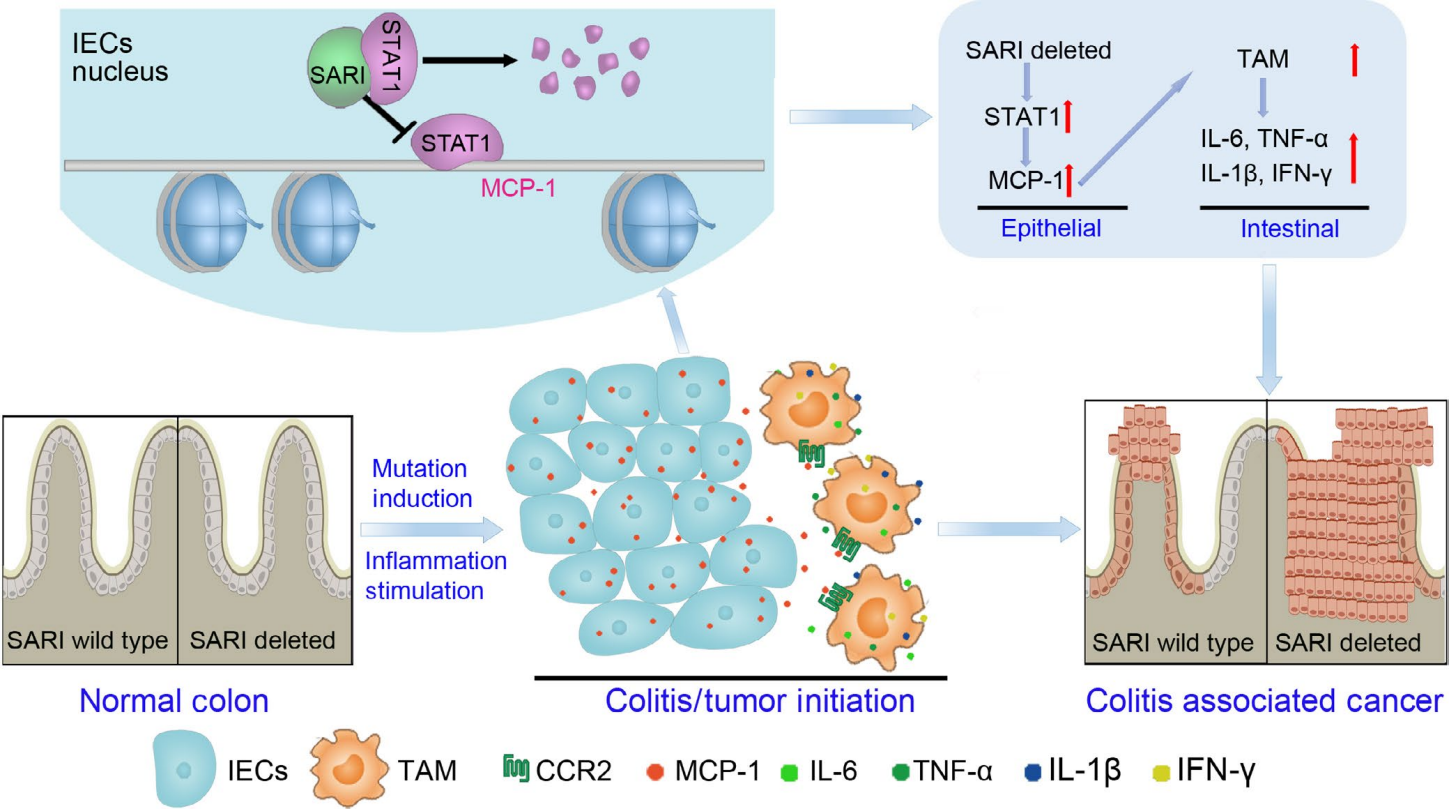
Molecular model of SARI regulating colitis and CAC. SARI inhibits colitis and CAC development by directly targeting and promoting protease‐dependent degradation of phosphorylated STAT1 and the transcriptional activity of *STAT1/MCP‐1*

According to the present findings and our previous study,^18^ we hypothesize that SARI impairs CAC through attenuating colitis by directly targeting and promoting protease‐dependent degradation of STAT1 and the transcriptional activity of STAT1/MCP‐1 (Figure 8).

## 4 DISCUSSION

Our results have shown that SARI deficiency promotes colon tumorigenesis only in the presence of colon inflammation. Further investigations suggested that more TAMs were infiltrated in the colon tissues of SARI deficiency mice during CAC development and MCP‐1‐mediated CCR2^+^ TAMs play a necessary role during SARI regulating tumour‐associated inflammation microenvironment and CAC development. Nuclear STAT1 was directly targeted and inhibited by SARI in colon cancer cells, which mediated the inhibition of MCP‐1 expression by SARI.

In agreement with previous evidence for the tumour suppressor role of SARI in mouse models of colon and other cancers,^15^, ^19^, ^20^ we showed that SARI deficiency enhances intestinal tumorigenesis in an inflammation‐dependent model. AOM is a chemical agent that initiates cancer via facilitating base mispairings,^21^ whereas DSS is a pro‐inflammatory reagent for mimicking chronic colitis‐associated tumour development when combined with AOM.^21^ Notably, the same factor may play distinct roles in AOM‐induced intestinal tumorigenesis and in the AOM/DSS‐induced CAC model. For example, knockout of IKKβ in intestinal mesenchymal cells (IMCs) impairs colitis‐associated tumorigenesis.^22^ Similarly, we showed that SARI deficiency promotes colitis‐associated tumorigenesis and causes higher mortality in mice, whereas no significant differences in tumour initiation and progression were found in colitis‐independent tumorigenesis model with SARI^WT^ and SARI^−/−^ mice. In addition, we found enhanced DSS‐induced inflammation and epithelial damage in SARI^−/−^ mice in the CAC model. Therefore, deletion of SARI promotes intestinal tumorigenesis through regulating colon inflammation, but not spontaneous tumorigenesis.

In accordance with its tumour suppressor role, SARI was found to be down‐regulated in many types of cancers,^15^, ^19^, ^23^, ^24^ but expressed at a high level in the normal colon, spleen, pancreas and prostate tissues.^12^ A previous study by our group indicated that the down‐regulation of SARI inversely correlates with poor clinical outcomes in colon cancer patients.^15^ In the present study, a decrease in intestinal SARI mRNA and protein expression accompanied the DSS treatment in the CAC model. Interestingly, injection of AOM significantly enhanced SARI expression both at the mRNA and protein level. We only observed the phenomenon of SARI deregulation during AOM/DSS‐induced intestinal tumorigenesis, and further investigations are needed to elucidate the underlying mechanism and epigenetic regulation involved in this process, as indicated in colon cancer.^16^

Various evidence has demonstrated the pro‐oncogenic role of macrophages by the phenotype of promoting immunosuppressive tumour microenvironment formation.^9^, ^10^ The previous study by us indicated that more extensive macrophage infiltration into colon sections in *SARI* ^−/−^ mice compared with that in *SARI* ^WT^ mice in acute colitis. In accordance with this, we also proved the inhibitional role of SARI in TAM infiltration into colon sections during CAC development, accompanied with elevated expression of iNOS in the macrophages. As an important factor of macrophage recruitment, MCP‐1 was up‐regulated in various cancers and promoted cancer development and metastasis.^25^, ^26^, ^27^ Thus, targeting MCP‐1, and followed with the recruitment of TAM is a potential strategy against diverse cancers, including colon cancer.^26^, ^28^, ^29^ Our results confirmed the up‐regulation of MCP‐1 in *SARI* ^−/−^ mice compared with that in *SARI* ^WT^ mice at different time points during CAC development, accompanied with recruitment of CCR2 positive TAM in colon tissues. CCR2 knockout blocks the SARI deficiency‐promoted tumorigenesis. Furthermore, STAT1 was also proved as the direct target of SARI in colon cancer cells, evidenced by Co‐IP and IF staining, as indicated in intestinal epithelial cells.^18^ Other transcriptional regulation factors of MCP‐1, including NF‐κB^30^, ^31^ and STAT3,^32^, ^33^ were up‐regulated in colon tissues of *SARI* ^−/−^ mice in CAC, but demonstrated not to be the direct target of SARI in colon cancer cells. The inverse correlation between SARI and p‐STAT1 was also observed in colon malignant tissues of patients, whereas SARI mRNA expression was positively correlated with STAT1 mRNA expression. This may contribute to the transcriptional regulation role of INF‐β both on SARI^12^ and p‐STAT1.^34^ Whether there is a dynamic regulation between SARI and STAT1 should be determined in further study.

In summary, the present results clarify a regulatory mechanism controlling tumour‐associated inflammation microenvironment during primary tumour development in the gastrointestinal tract (Figure 8). Our study may help identify new therapeutic approaches to control intestinal tumorigenesis, which might be relevant for other inflammation‐associated cancers.

## CONFLICT OF INTEREST

All authors declare that they have no conflicts of interest.

## AUTHOR CONTRIBUTIONS

Lei Dai, Yi Liu, Junshu Li, Na, Chen, Lin Cheng, Huiling Wang, Yi Lin, Gang Shi and Zhexu Dong were involved in the acquisition of data. Lei Dai and Hongxin Deng were involved in study concept design, analysis and interpretation of data. Lei Dai was involved in drafting of the manuscript. Hongxin Deng was involved in critical revision of the manuscript for important intellectual content. Hongxin Deng and Lei Dai were involved in obtained funding. Yuan Yin, Chao Fang, Ping Fan, Lie Yang, Wei Huang and Zongguang Zhou were involved in human colon cancer tissues collected. Hantao Zhang and Xiaolan Su were involved in animal study. Shuang Zhang and Yang Yang were involved in technical support and analysis and interpretation of data. Dechao Yu was involved in obtained funding and study supervision.
