# Supplementary material for: SARI suppresses colitis‐associated cancer development by maintaining MCP‐1‐mediated tumour‐associated macrophage recruitment
Source: J Cell Mol Med. 2019 Oct 2;24(1):189–201. doi: 10.1111/jcmm.14699 (PMC6933368; doi:10.1111/jcmm.14699)
Supplement: Supplementary file 1 [file JCMM-24-189-s001.docx]

**
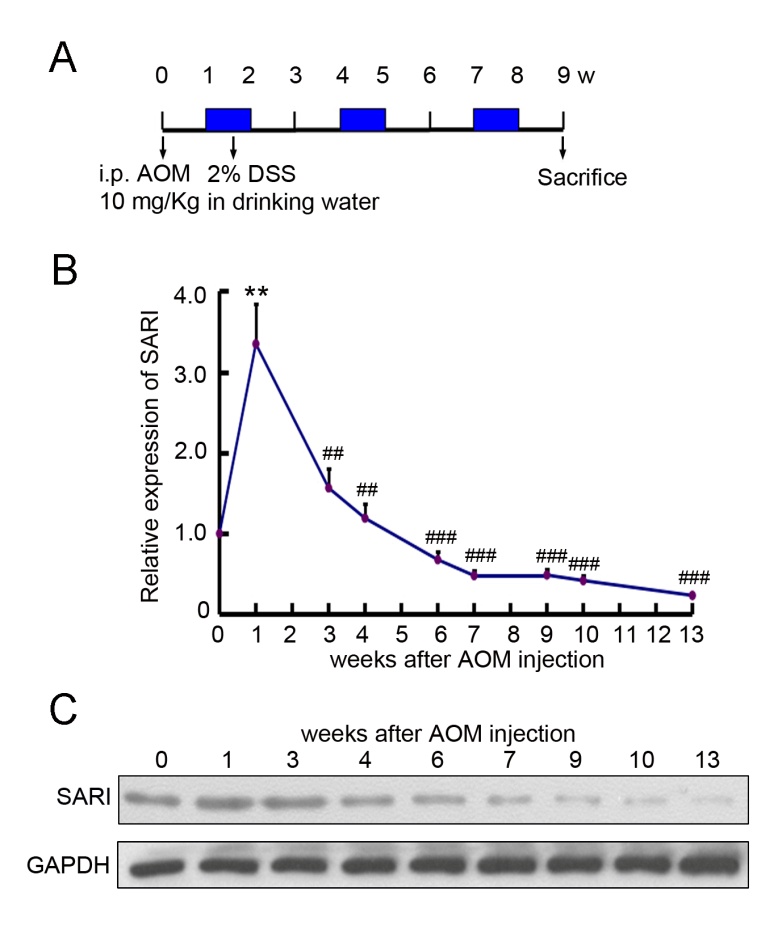
**

**Supplemental Figure 1**

(A) Schematic diagram of the AOM/DSS model of CAC. A single AOM injection (10 mg/kg) is followed by three cycles of 2% DSS administration in the drinking water.

(B) qPCR analysis of colonic SARI expression from AOM/DSS model of CAC mice. Data represent means ± SD, analysis of variance. **, p<0.01, compared with 0 time point; ##, p<0.01, ###, p<0.001, compared with 1 time point.

(C) Western blotting analysis of colonic SARI expression from AOM/DSS model of CAC mice.

**
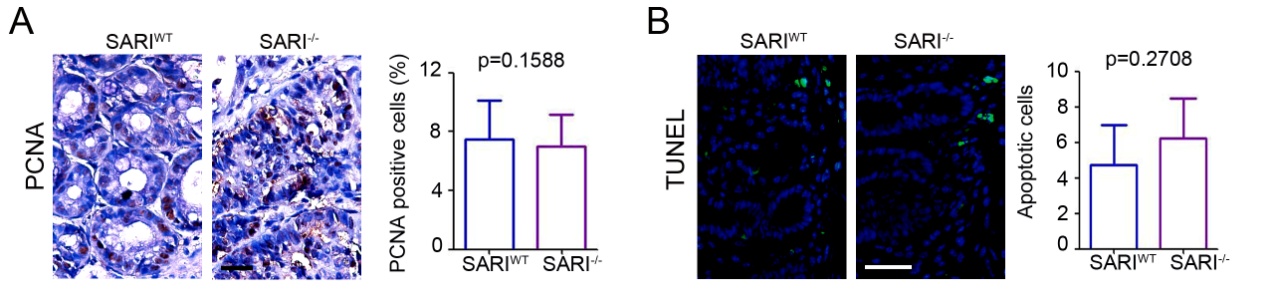
**

**Supplemental Figure 2**

Representative images of CD68 staining of colon malignant, adjacent and forward tissues from patients. The expression of CD68 was scored. n=20, Scale bar = 200 μm. Data represent means ± SD, Student’s *t*-test.

**
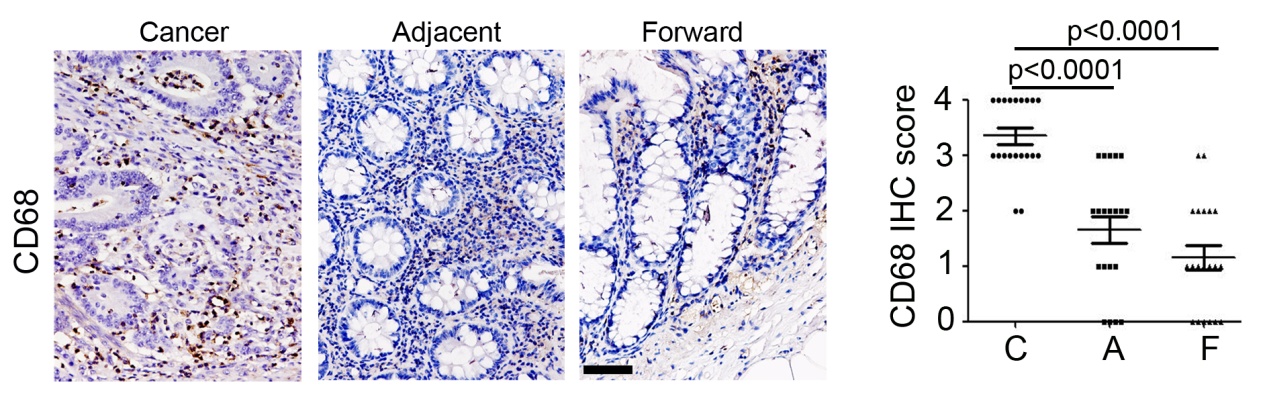
**

**Supplemental Figure 3**

(A) The mortality rate of SARI^WT^ mice with SARI^WT^ BM (BM^WT^mice^WT^), SARI^-/-^ mice with SARI^-/-^ BM (BM^-/-^ mice^-/-^), SARI^WT^ mice with SARI^-/-^ BM (BM^-/-^mice^WT^) and SARI^-/-^ mice with SARI^WT^ BM (BM^-/-^mice^WT^) receiving AOM/DSS treatment.

(B) Representative images of PCNA staining of colon tumors at the end of the AOM/DSS treatment. The percent of PCNA positive cells were microscopically analyzed. Scale bar = 50 μm, **, p<0.05; ns, no significant difference. Data represent means ± SD, analysis of variance.

**
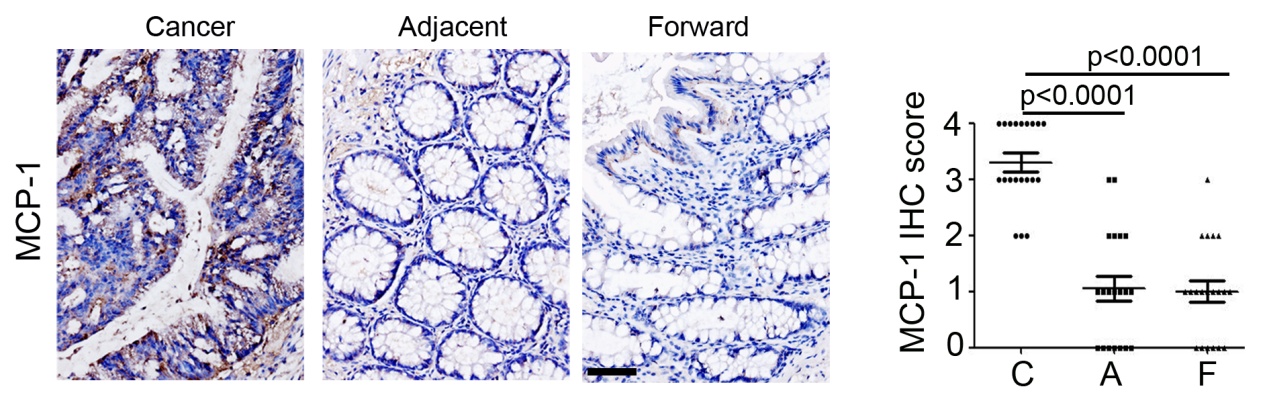
**

**Supplemental Figure 4**

Representative images of MCP-1 staining of colon malignant, adjacent and forward tissues from patients. The expression of MCP-1 was scored. n=20, Scale bar = 200 μm. Data represent means ± SD, analysis of variance.

**
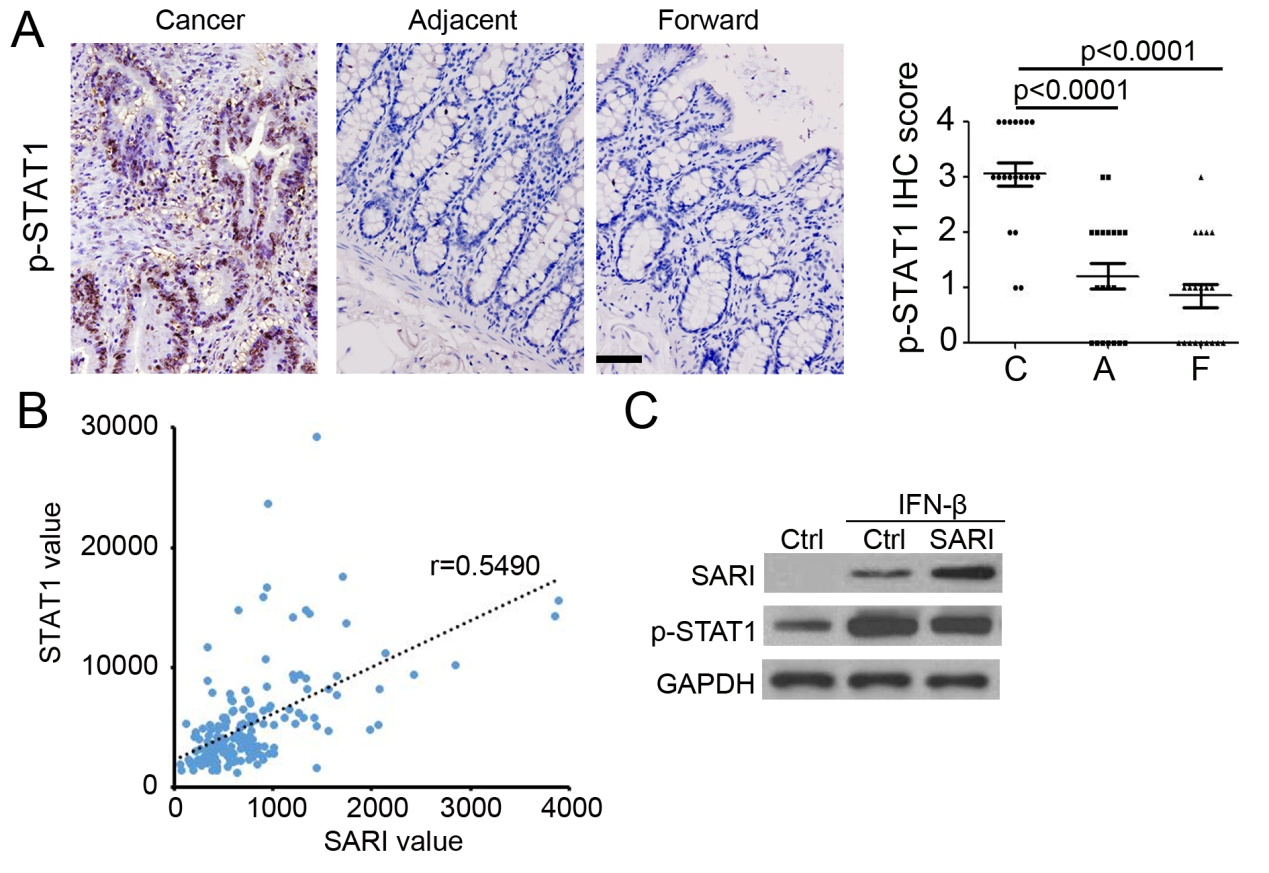
**

**Supplemental Figure 5**

(A) Representative images of p-STAT1 staining of colon malignant, adjacent and forward tissues from patients. The expression of p-STAT1 was scored. n=20, Scale bar = 200 μm. Data represent means ± SD, analysis of variance.

(B) The correlation analysis of SARI mRNA and STAT1 mRNA expression in clinical samples(n=176) from colon cancer patients. Pearson correlation analysis.

(C) Immunoblots of p-STAT1 expression in SW480 cells with or without IFN-β treatment (0.01 μg/ml).
